# Effect of quinolinic acid-induced lesions of the subthalamic nucleus on performance on a progressive-ratio schedule of reinforcement: A quantitative analysis

**DOI:** 10.1016/j.bbr.2008.09.005

**Published:** 2008-12-22

**Authors:** G. Bezzina, F.S. den Boon, C.L. Hampson, T.H.C. Cheung, S. Body, C.M. Bradshaw, E. Szabadi, I.M. Anderson, J.F.W. Deakin

**Affiliations:** a Psychopharmacology Section, Division of Psychiatry, University of Nottingham, Room B109, Medical School, Queen’s Medical Centre, Nottingham NG7 2UH, UK; b Neuroscience & Psychiatry Unit, School of Psychiatry & Behavioural Sciences, University of Manchester, Stopford Building, Oxford Road, Manchester M13 9PT, UK

**Keywords:** Subthalamic nucleus, Lesion, Quinolinic acid, Progressive-ratio schedule, Reinforcer efficacy, Rat

## Abstract

The subthalamic nucleus (STN), a major relay in the indirect striatofugal pathway, plays an important role in extrapyramidal motor control. Recent evidence indicates that it may also be involved in regulating the incentive value of food reinforcers.

**Objective:**

To examine the effect of lesions of the STN on performance on a progressive-ratio schedule using a quantitative model that dissociates effects of interventions on motor and motivational processes [Killeen PR. Mathematical principles of reinforcement. Behav Brain Sci 1994;17:105–72]. Rats with bilateral quinolinic acid-induced lesions of the STN (*n* = 14) or sham lesions (*n* = 14) were trained to press a lever for food-pellet reinforcers under a progressive-ratio schedule. In Phase 1 (90 sessions) the reinforcer was one pellet; in Phase 2 (30 sessions) it was two pellets; in Phase 3 (30 sessions) it was again one pellet.

**Results:**

The performance of both groups conformed to the model of progressive-ratio schedule performance. The motor parameter, *δ*, was significantly higher in the STN-lesioned than the sham-lesioned group, reflecting lower overall response rates in the lesioned group. The motivational parameter, *a*, was significantly higher in the STN-lesioned group than in the sham-lesioned group, consistent with enhanced reinforcer value in the STN-lesioned group compared to the sham-lesioned group. In both groups, *a* was sensitive to changes in reinforcer size, being significantly greater under the two-pellet condition (Phase 2) than under the one-pellet condition (Phases 1 and 3). The results suggest that destruction of the STN impairs response capacity and enhances the incentive value of food reinforcers.

## 1 Introduction

The subthalamic nucleus (STN) is an important component structure of the cortico-striato-thalamo-cortical circuitry that is believed to regulate a wide range of motor and cognitive functions [2,22,37,47]. Lesions of the STN have been found to reverse experimentally induced rigidity and bradykinesia in animal models of Parkinson’s disease [12–14], and inactivation of the STN by high-frequency stimulation can have a similar beneficial effect in patients suffering from this disease [34,35].

Recently there has been increasing interest in the possible role of the STN in motivational processes. Destruction of the STN has been found to promote premature responding in serial reaction time tasks [3,8,9] and differential-reinforcement-of-low-response-rate (DRL) schedules [49]. These effects have been attributed to enhancement of ‘impulsive action’, in other words to the release of positively reinforced behaviour from learned inhibitory control [8,39; for review, see ref. 47]. In addition, recent evidence suggests that some behavioural effects of lesions of the STN may be brought about by an enhancement of the incentive value of reinforcers. For example, destruction of the STN has been found to increase the locomotor activation induced by conditioned stimuli that have been associated with food reward [10], and to prolong responding on progressive-ratio schedules of food [11] and cocaine [48] reinforcement.

The aim of the experiment described in this paper was to provide further information about the effect of lesions of the STN on performance on progressive-ratio schedules. We used a mathematical approach to analyse performance on progressive-ratio schedules [30] in order to address the question of whether destruction of the STN causes an enhancement of the incentive value of a food reward.

In a progressive-ratio schedule [25,26], the number of responses required to earn a reinforcer (the response/reinforcer ratio) increases progressively with successive reinforcers. The traditional measure of performance on this schedule is the ratio at which responding ceases for some pre-defined period (the ‘breakpoint’: [6,25,45], or alternatively the highest ratio completed within a time-limited experimental session [1,23,24,51]. The breakpoint has traditionally been regarded as an index of the subject’s motivational state [7,19,21], or the incentive value of the reinforcer [20,25], an interpretation that has been supported by reports that the breakpoint is sensitive to changes in deprivation level and reinforcer size [21,43,44]. There are, however, significant problems with the use of the breakpoint as an index of motivation or reinforcer value. For example, this parameter has been shown to be sensitive to ‘non-motivational’ manipulations such as changes in the response requirement [1,43,46] and the ratio step size [33,45]. The breakpoint also suffers from the weakness that it is derived from a single time point during an experimental session, the data obtained during the rest of the session being ignored [4,33].

One way of circumventing these difficulties is the application of Killeen’s quantitative model of ratio-schedule performance [30,31] which takes into account the response rate in each component ratio of the schedule. This model is derived from Killeen’s general theory of schedule-controlled behaviour, the Mathematical Principles of Reinforcement (MPR) [30], which is founded on fundamental postulates about the incentive value of reinforcers, biological constraints on responding, and the efficiency with which particular reinforcement schedules couple operant responses to reinforcers. In the case of ratio schedules, in which *N* responses are required for each reinforcer delivery, response rate, *R*, is predicted by

(1) $$R=\frac{\zeta }{\delta }-\frac{N}{a},\;\text{where}\;\zeta =1-{\left(1-\beta \right)}^{N};\;a,\;\delta >0;\;0<\beta <1\text{.}$$

The parameter β (‘currency’) represents the extent to which the strengthening effect of the reinforcer is focussed on the most recent response, *δ* (‘response time’) is the reciprocal of the maximum response rate, and *a* (‘specific activation’) is the time for which a reinforcer is able to activate behaviour. The last of these parameters, *a*, provides an index of reinforcer efficacy or ‘value’ [30,32,41]. Consistent with the interpretation of *a* as an index of reinforcer value, it has been demonstrated that this parameter is sensitive to manipulation of reinforcer size and quality [16,17,41]. Reilly [41] has recommended the use of *a* to construct a quantitative scale of reinforcer value. Although Eq. (1) was originally proposed as a model of fixed-ratio performance [30], it also provides a good description of performance on progressive-ratio schedules, and has been used to evaluate the effects of centrally acting drugs [24,36,41,53,54] and cerebral lesions [16,29] on reinforcer efficacy.

In this experiment we examined the effect of lesions of the STN on the parameters of Eq. (1). Based on the supposition that destruction of the STN results in enhancement of reinforcer value [11,48], it was predicted that STN-lesioned rats would exhibit higher values of the parameter *a* than intact (sham-lesioned) rats.

## 2 Methods

The experiment was carried out in accordance with UK Home Office regulations governing experiments on living animals.

### 2.1 Subjects

Thirty experimentally naive female Wistar rats approximately 4 months old and weighing 250–300 g at the start of the experiment were used. They were housed under a constant cycle of 12 h light and 12 h darkness (light on 0600–1800 h), and were maintained at 80% of their initial free-feeding body weights throughout the experiment by providing a limited amount of standard rodent diet after each experimental session. Tap water was freely available in the home cages.

### 2.2 Surgery

The rats received either lesions of the STN (*n* = 16) or sham lesions (*n* = 14). Anaesthesia was induced with isoflurane (4% in oxygen), and the rat positioned in a stereotaxic apparatus (David Kopf), with the upper incisor bar set 3.3 mm below the inter-aural line. Anaesthesia was maintained with 2% isoflurane in oxygen during surgery. A small hole was drilled in the skull over each hemisphere for microinjection of quinolinic acid into the STN. The following coordinates were used to locate the STN: AP −3.6, L ±2.6, V −8.0 (mm, measured from bregma [38]). Injections were given via a 0.3 mm diameter cannula connected by a polyethylene tube to a 10-μl Hamilton syringe. In the case of the lesioned group, the cannula tip was slowly lowered to the position of each site and 0.3 μl of a 0.1-M solution of quinolinic acid (2,3-pyridinedicarboxylic acid) in phosphate-buffered 0.9% NaCl (pH 7.0) was injected at a rate of 0.1 μl per 15 s. The cannula was left in its position for 3 min after completion of the injection in each site. In the case of the sham-lesioned group, the procedure was identical, except that the vehicle alone was injected. The rats were given diazepam 5 mg kg^−1^ intraperitoneally in order to suppress seizures during the immediate post-operative period.

### 2.3 Apparatus

The rats were trained in operant conditioning chambers of internal dimensions 20 cm × 23 cm × 22.5 cm (Campden Instruments Ltd.). One wall of the chamber contained a recess into which a motor-operated dispenser could deliver food pellets (TestDiet, MLab Rodent Tablet 45 mg; Sandown Scientific, UK). An aperture was situated 5 cm above and 2.5 cm to one side of the recess (left for half the rats, right for the other half); a motor-operated retractable lever could be inserted into the chamber through this aperture. The lever could be depressed by a force of approximately 0.2 N. The chamber was enclosed in a sound-attenuating chest; masking noise was provided by a rotary fan. An Acorn 5000 microcomputer and interface unit (Paul Fray Ltd.), programmed in ARACHNID BASIC and located in an adjoining room, controlled the schedules and recorded the behavioural data.

### 2.4 Behavioural training

Two weeks after surgery, the food deprivation regimen was introduced and the rats were gradually reduced to 80% of their free-feeding body weights. Then they were trained to press the lever for a food-pellet reinforcer (45 mg), and were exposed to a fixed-ratio 1 schedule for 3 days, followed by a fixed-ratio 5 schedule for 3 days. Thereafter, they underwent daily training sessions under the progressive-ratio schedule. The progressive-ratio schedule was based on the following exponential progression: 1, 2, 4, 6, 9, 12, 15, 20, 25, 32, 40, 50, …, derived from the formula [(5 × e^0.2*n*^) − 5], rounded to the nearest integer, where *n* is the position in the sequence of ratios [42]. Sessions took place at the same time each day during the light phase of the daily cycle (between 0800 and 1400 h) 7 days a week. At the start of each session, the lever was inserted into the chamber; the session was terminated by withdrawal of the lever 50 min later. The experiment consisted of three phases: in phase 1 (90 sessions) the reinforcer was a single 45-mg food pellet, in phase 2 (30 sessions) it was 2 pellets, and in phase 3 (30 sessions) it was again one pellet.

### 2.5 Histology

At the end of the behavioural experiment, the rats were deeply anaesthetised with sodium pentobarbitone, and perfused transcardially with 0.9% sodium chloride, followed by 10% formol saline. The brains were removed from the skull and fixed in formol saline for 1 week. 40-μm coronal sections were taken through the region of the STN using a freezing microtome.

#### 2.5.1 Cresyl violet staining

The procedure was similar to that described previously [28]. Alternate sections were mounted on chrome-gelatine-coated slides and air dried, hydrated by successive immersion in 95%, 70% and 50% ethanol, stained in 0.25% cresyl violet for 2 min at room temperature, dehydrated by successive immersion in 50%, 70%, 95%, 100% ethanol and xylene, and mounted with DPX.

#### 2.5.2 Immunocytochemistry

In the other sections neurone-specific nuclear protein (NeuN) was labelled as described by Jongen-Relo and Feldon [27]. Our protocol has been described elsewhere [15]. Briefly, freshly sliced sections were rinsed in 0.1 M phosphate-buffered saline (PBS) and placed in 0.5% H_2_O_2_ in PBS for 30 min. After twice rinsing in PBS, they were placed for 1 h in a blocking solution (10% normal horse serum [Vector Laboratories, Peterborough, UK], 1% bovine serum albumin [BSA, Sigma–Aldrich, Gillingham, UK] and 0.3% Triton X-100 [Sigma–Aldrich] in PBS). They were incubated for 48 h at 4 °C with the primary antibody (monoclonal mouse anti-NeuN serum [1:5000, Chemicon, Chandlers Ford, UK] in 1% normal horse serum, 1% BSA and 0.3% Triton X-100 in PBS), washed twice in PBS, and incubated for 2 h at room temperature in biotinylated horse antimouse serum (Vector Laboratories) (1:1000 in 1% BSA and 0.3% Triton X-100 in PBS). After further rinsing in PBS, they were placed for 2 h in avidin-biotin-horseradish peroxidase complex (1:200, ABC-Elite, Vector Laboratories) in PBS. After two further rinses in PBS, they were placed in a chromagen solution (0.05% diaminobenzidine [Sigma–Aldrich] and 0.01% H_2_O_2_ [Sigma–Aldrich]) for 5 min. The reaction was observed visually and stopped by rinsing in PBS. The sections were floated on to chrome-gelatine-coated slides and mounted with DPX.

An investigator who was blind to the behavioural results performed the microscopic examination. Drawings of the area of the lesions were superimposed on the appropriate coronal sections in the stereotaxic atlas of Paxinos and Watson [38].

### 2.6 Data analysis

Data from two rats in the lesioned group were discarded because the lesions were found to be misplaced, leaving 14 rats in each group. Mean data derived from the last 10 sessions of each phase of the experiment were used in the statistical analyses.

#### 2.6.1 Raw data

##### 2.6.1.1 Highest completed ratio and peak response rate

The breakpoint was defined as the last ratio to be completed before 5 min elapsed without any responding [25,26]. In most cases, this was identical to the highest ratio completed in the session. However, in some cases, the breakpoint criterion was not met within the 50 min session. Therefore the highest completed ratio, rather than breakpoint, was adopted as the performance measure for analysis. The highest completed ratio and the peak response rate (i.e. the highest overall response rate, corresponding to the peak of the response-rate function; see Fig. 2) were analysed by two-factor analyses of variance (group × phase).

##### 2.6.1.2 Overall response rate

Overall response rate was calculated for each ratio using the total time taken to complete the ratio, including the post-reinforcement pause, measured from the end of the preceding reinforcer delivery until the emission of the last response of the ratio [17]. The first ratio (a single response) and any ratios that had not been completed at the end of the session were excluded from the analysis. The data were analysed by three-factor analysis of variance (group × phase × ratio), with repeated measures on the second and third factors. The data were subjected to function-fitting and statistical analysis as described below.

##### 2.6.1.3 Post-reinforcement pause

The post-reinforcement pause was defined as the time from the end of the preceding reinforcer delivery until the first response of the ratio. These data were analysed by three-factor analyses of variance (group × phase × ratio), with repeated measures on the second and third factors.

##### 2.6.1.4 Running rate

Running rate, calculated by dividing the number of responses by the ‘run-time’ (i.e. the time taken to complete the ratio, excluding the post-reinforcement pause [18]), was analysed by three-factor analysis of variance (group × phase × ratio), with repeated measures on the second and third factors.

Because the number of ratios completed within a session under a progressive-ratio schedule differs among individual subjects, analyses of variance of the raw response rates and post-reinforcement pauses included only those ratios that were completed by at least 75% of the rats in each group in each phase of the experiment (ratios up to and including 62), missing values being filled using the value obtained in the highest ratio completed by the subject in question (this was felt to be a more conservative approach to replacing missing data than estimation based on extrapolation). Missing data replaced in this way amounted to 8 out of a total of 336 data points in each analysis. Note that the quantitative analysis (see below) was not subject to this limitation, because Eq. (1) was fitted to the data from individual subjects.

#### 2.6.2 Quantitative analysis

Eq. (1) was fitted to the overall response rate data obtained from each rat using an iterative least-squares method (SigmaPlot, Version 8.0), and the estimated values of the parameters β, *δ* and *a* were derived; goodness of fit was expressed as *r*^*2*^, the proportion of the data variance accounted for by the equation. In agreement with previous findings [16,24,36,53,54] examination of the data revealed that in some rats very low response rates were generated under the highest ratios, which did not conform to Eq. (1). Therefore the equation was fitted to each rat’s data after exclusion of these low rates using the following operational criterion [24,36]. Points were removed successively, starting from the highest ratio completed, when the curve-fitting routine generated an abscissa intersection point (*a*/*δ*) which lay to the left of the rightmost empirical datum point; such an intersection implies a negative predicted response rate, which is impossible empirically, and specifically precluded by the model (see above, Eq. (1)). A fit was accepted when the predicted response rates for all the surviving data points had positive values. This procedure seldom eliminated more than one datum point from the data sets derived from individual rats (see Section 3). The estimates of each parameter were compared across phases using the data obtained from the final 10 sessions of each phase using two-factor analyses of variance (group × phase) with repeated measures on the second factor, followed, if appropriate, by multiple comparisons within each phase.

A significance criterion of *P* < 0.05 was adopted in all statistical analyses.

## 3 Results

### 3.1 Behavioural data

#### 3.1.1 Raw data

##### 3.1.1.1 Highest completed ratio

**Fig. 1:**
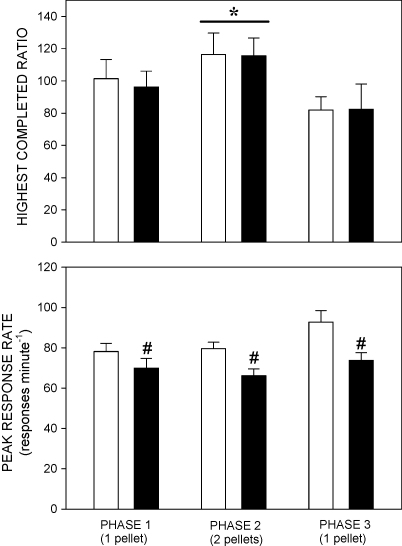
Upper histogram: highest completed ratio. Lower histogram: peak response rate (responses min^−1^). Bars show group mean data ± SEM, for the sham-lesioned group (white) and the STN-lesioned group (black), in each phase of the experiment. * Significantly higher ratio in both groups in phase 2 than in phases 1 and 3 (*P* < 0.05); ^#^ Significantly lower peak response rate in the STN-lesioned group than in the sham-lesioned group in all three phases of the experiment (see text for details).

The group mean highest completed ratios (±SEM) in the three phases of the experiment are shown in Fig. 1 (upper histogram). The analysis of variance revealed a significant main effect of phase [*F*(2,52) = 22.1, *P* < 0.001], reflecting the attainment of higher ratios by both groups in phase 2, when the reinforcer consisted of two pellets, than in phases 1 and 3, when it consisted of a single pellet. There was no significant main effect of group and no significant group × phase interaction [*F*s < 1].

##### 3.1.1.2 Peak response rate

Peak response rates are shown in Fig. 1 (lower histogram). The main effect of group was significant [*F*(1,26) = 6.8, *P* < 0.02], reflecting higher peak rates in the sham-lesioned than the STN-lesioned group; the effect of phase was also significant [*F*(2,52) = 9.0, *P* < 0.001], reflecting the tendency for peak rate to be higher in phase 3 than in phases 1 and 2; the group × phase interaction was not significant [*F*(2,52) = 2.0, NS].

##### 3.1.1.3 Overall response rate

**Fig. 2:**
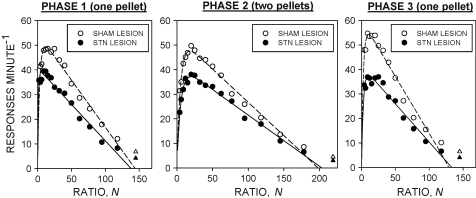
Overall response rates under the progressive-ratio schedule during the final 10 sessions of each phase of the experiment. Ordinate: response rate (responses min^−1^); abscissa*:* response/reinforcer ratio. Points are group mean data from the sham-lesioned (open symbols) and STN-lesioned (filled symbols) groups. The curves are fits of Eq. (1) to the data. See text for details of analysis. Triangles are data points excluded from the function-fitting (see text for explanation).

Fig. 2 shows the group mean overall response rates as a function of the response-reinforcer ratio in each phase of the experiment; the curves are the functions defined by Eq. (1) (see below). Response rates tended to be lower in the STN-lesioned group than in the sham-lesioned group in the lower ratios, this tendency becoming less apparent as the ratio increased. Analysis of variance revealed significant main effects of group [*F*(1,26) = 6.2, *P* < 0.02] and ratio [*F*(11,286) = 11.4, *P* < 0.001], but not of phase [*F* = 2.1, NS]. There were significant group × phase [*F*(2,52) = 3.6, *P* < 0.05] and phase × ratio [*F*(22,572) = 8.8, *P* < 0.001] interactions. The group × ratio interaction [*F* < 1] and the group × phase × ratio interaction [*F* = 1.0, NS] were not statistically significant.

##### 3.1.1.4 Post-reinforcement pause

**Fig. 3:**
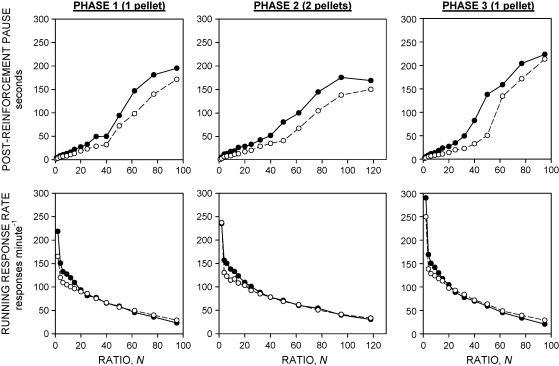
Performance in successive ratios of the progressive-ratio schedule during the final 10 sessions of each phase of the experiment. Upper panels: Post-reinforcement pause, ordinate, pause duration (s). Lower panels: Running response rate (response rate calculated after exclusion of the post-reinforcement pause), ordinate, running rate (responses min^−1^). Other conventions are as in Fig. 1.

The upper panels of Fig. 3 show the relationship between post-reinforcement pause and the ratio requirement in the three phases of the experiment. Analysis of variance revealed significant main effects of group [*F*(1,26) = 5.6, *P* < 0.05] and ratio [*F*(11,286) = 40.3, *P* < 0.001] and significant group × ratio [*F*(11,286) = 1.9, *P* < 0.05] and phase × ratio [*F*(22,572) = 2.5, *P* < 0.01] interactions. Neither the main effect of phase [*F*(2,52) = 2.6, NS] nor the three-way interaction [*F*s < 1] was statistically significant.

##### 3.1.1.5 Running response rate

The lower panels of Fig. 3 show the running response rate data. Running response rate declined monotonically as a function of ratio in both groups. The STN-lesioned rats tended to show somewhat higher running response rates than the sham-lesioned rats at intermediate ratios (between 2 and 20). These findings are reflected in the results of the analysis of variance: there were significant main effects of phase [*F*(2,52) = 29.4, *P* < 0.001] and ratio [*F*(11,286) = 160.3, *P* < 0.001], but not of group [*F*(1,26) = 1.2, NS]. There was no significant phase × group interaction [*F* < 1], but the ratio × group [*F*(11,286) = 3.0, *P* < 0.01] and phase × ratio × group [*F*(22,572) = 1.6, *P* < 0.05] interactions were statistically significant.

#### 3.1.2 Quantitative analysis: parameters of Eq. (1)

The fits of Eq. (1) to the group mean overall response rates in each phase of the experiment are shown in Fig. 2. In each case the function accounted for more than 95% of the variance of the group mean data of each group (*r*^2^ > 0.95). Eq. (1) was also fitted to the overall response-rate data obtained from the individual rats in each group. The values of the parameters derived in the final 10 sessions of each phase are shown in Table 1.

Specific activation (*a*): Analysis of variance showed a significant effect of group [*F*(1,26) = 4.6, *P* < 0.05], reflecting the consistently higher values of the parameter in the STN-lesioned group than in the sham-lesioned group. There was a significant effect of phase [*F*(2,52) = 12.7, *P* < 0.001], reflecting the increase in the value of this parameter when the reinforcer size was increased from one pellet to two. There was no significant interaction [*F*(2,54) = 2.0, NS]. Analysis of the simple main effects showed that the effect of phase was significant both in the sham-lesioned group [*F*(2,26) = 7.1, *P* < 0.01] and in the STN-lesioned group [*F*(2,26) = 6.8, *P* < 0.01]. The increase in *a* brought about by the introduction of the larger reinforcer size in phase 2, compared to phases 1 and 3, was 52.7% (±12.5%) in the sham-lesioned group and 66.4% (±11.8%) in the STN-lesioned group.

Response time (*δ*) was significantly higher in the STN-lesioned group than in the sham-lesioned group [*F*(1,26) = 7.7, *P* < 0.05], indicating a lower maximum response rate in the STN-lesioned group. There was no significant main effect of phase [*F*(2,52) = 2.9, NS], and no significant group × phase interaction [*F* < 1].

The currency parameter (β) showed no significant main effect of group [*F* < 1] or phase [*F*(2,52) = 3.1, NS], and the group × phase interaction [*F* < 1] was not significant.

Table 1 also shows the mean (±SEM) number of data points excluded from fitting of Eq. (1) to the data from individual rats in each phase of the experiment. The overall number of excluded data points amounted to 1.98% of the entire data set.

### 3.2 Histology

**Fig. 4:**
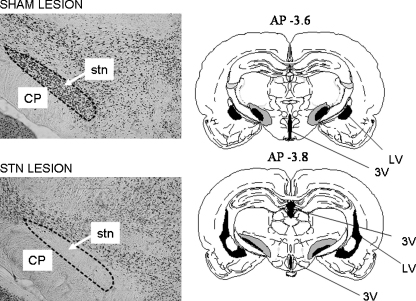
Left-hand panels: sample photomicrographs from NeuN-stained coronal sections of the brains of a sham-lesioned rat (upper panel) and an STN-lesioned rat (lower panel). CP: cerebral peduncle. The dotted outline shows the extent of the STN in the sham-lesioned rat. Note the cell loss in the STN in the lesioned rat. Right-hand diagrams: representation of the approximate area of destruction of the STN in the lesioned group. Drawings were made from the microscopic sections, and were superimposed on the relevant pages from Paxinos and Watson’s [38] stereotaxic atlas. The black area in the region of the STN represents the smallest, and the surrounding grey area the largest extent of the lesion. LV: lateral ventricle; 3V: third ventricle.

Bilateral lesions were found to be accurately placed in 14 of the 16 rats that had received injections of quinolinic acid into the STN (the behavioural data from the remaining two rats were excluded from all analyses: see above). There was marked neuronal loss in the STN compared to the sham-lesioned rats. Neuronal loss was mainly restricted to the STN, although some minor loss was seen in the zona incerta and lateral hypothalamus immediately adjacent to the STN in some animals. Examples of NeuN-labelled sections are shown in the left-hand panels of Fig. 4; the approximate extent of the lesion is shown in the right-hand diagrams.

## 4 Discussion

Injection of quinolinic acid produced a substantial lesion of the STN, of approximately the same extent as those seen in previous experiments using similar surgical protocols with the excitotoxin ibotenic acid [11,48,49]. The STN was almost completely destroyed in most of the lesioned rats.

The performance of both groups on the progressive-ratio schedule was qualitatively similar to that reported in many previous studies [e.g. 5,16,17,24,29,33,36,53,54]. Overall response rate was bitonically related to ratio size, initially rising to a peak and then declining as a function of increasing ratio size. Post-reinforcement pause increased monotonically, and running response rate declined monotonically, as a function of ratio size. The increase in reinforcer size from one food pellet to two during phase 2 resulted in a significant increase in the highest completed ratio, consistent with previous reports [16,43,44]. The STN lesion did not significantly alter the highest completed ratio in this experiment. This is not necessarily in contradiction with previous reports that STN lesions result in an increase in the breakpoint in progressive-ratio schedules [11,48], because unlike those previous experiments the present experiment employed a time-limited session (50 min); it is possible that the use of a longer session would have revealed an increase in the breakpoint.

As in previous experiments, overall response rates in the progressive-ratio schedule showed good conformity to Eq. (1) [16,24,33,36,53,54]. In phase 2, when the reinforcer size was increased from one food pellet to two, there was a significant increase in the ‘specific activation’ parameter, *a*. This is consistent with the predictions of MPR, according to which *a* reflects the efficacy of an individual reinforcer, and with previous findings both with pigeons [17] and with rats [16,18].

It has been noted previously that larger reinforcers tend to be associated with higher values of the ‘response time’ parameter, *δ*, possibly reflecting a greater contribution of post-prandial behaviour to post-reinforcement pauses in the case of larger reinforcers [16,17]. A similar trend was apparent in the present experiment, higher values of *δ* being seen in phase 2 (two-pellet condition) compared to phases 1 and 3 (one-pellet condition). However, in the present experiment, although there were significant differences in the peak response rate between the two- and one-pellet conditions, the corresponding differences in the value of *δ* did not achieve statistical significance.

The ‘currency’ parameter, β, was not significantly influenced by reinforcer size. In this respect, the present results differ from those of a previous experiment, in which an increase in reinforcer size resulted in a significant reduction of this parameter [16]. Bezzina et al. [16] noted that, according to MPR, β encapsulates the coupling of responses to reinforcers [17,30], and suggested that their finding might be explained in terms of a greater propensity of larger reinforcers to exert control over longer sequences of responses than smaller reinforcers. The reason for the discrepancy between the present results and Bezzina et al.’s [16] finding is unclear. Ongoing work in our laboratory suggests that β is sensitive to variation of reinforcer size; however relatively large differences in reinforcer size may be needed in order to elicit reliable effects.

The principal aim of the experiment was to examine the effects of destruction of the STN on the parameters of Eq. (1). The lesion produced a significant increase in the ‘activation’ parameter, *a*, this being reflected in the flatter slope of the descending limb of the response rate function in the STN-lesioned group compared to the sham-lesioned group (Fig. 2). Since, according to MPR, *a* yields a numerical index of the value of the food reinforcer, the present results provide quantitative confirmation of evidence from previous studies which indicated that destruction of the STN may enhance the reinforcing efficacy of positive reinforcers [10,47–49]. The precise nature of the enhancement of reinforcer efficacy is a subject of ongoing debate. The recent finding that STN lesions enhanced sign-tracking behaviour directed towards stimuli previously paired with positive reinforcers has led to the suggestion that the STN helps to regulate the amount of ‘incentive salience’ attributed to reward-related stimuli [49]. Although the present results are compatible with this interpretation, they do not exclude other interpretations. For example it has been proposed that *a* is a composite construct comprising an intrinsic motivational component as well as the incentive value of the reinforcer, suggesting that an increase in the value of this parameter might reflect either an increase in the palatability of the food reinforcer or an increase in deprivation-induced motivation [40].

The present experiment employed a food reinforcer. There does not appear to have been any previous attempt to apply MPR to pharmacological reinforcers. In view of existing evidence that STN lesions can enhance the incentive value of cocaine [48,50], it would be of interest in future experiments to examine the effect of STN lesions on progressive-ratio schedule performance maintained by drug reinforcers using this approach.

One advantage of the quantitative analysis used in the present experiment is that it allows the effects of an intervention on incentive value (represented by *a*) to be distinguished from effects on motor performance (represented by *δ*). The STN lesion produced an increase in *δ*, consistent with an impairment of motor performance. It should be noted that this does not necessarily imply that the lesion resulted in motor debility. Indeed, lesions of the STN do not impair, and may even facilitate locomotor behaviour [52]. Moreover, the data shown in Fig. 3 indicate that the reduction of the maximal overall response rate was not caused by an inability to respond rapidly, since the lesion produced an increase in post-reinforcement pausing, but did not impair running response rate. Interestingly, this pattern of effect differs from the effect of lesions of the nucleus accumbens core (AcbC), in which an increase in post-reinforcement pausing was accompanied by a reduction of running response rate [16].

The mechanisms whereby destruction of the STN can simultaneously produce an enhancement of incentive value and an impairment of motor performance remain to be determined. However, it is of interest to note that emerging anatomical evidence indicates that the motor and motivational functions of the STN may be represented by distinct subdivisions of the nucleus, the dorsolateral portion being mainly involved with extrapyramidal motor control and the medial (‘limbic’) portion with motivational functions [37,47]. The lesions inflicted in the present experiment presumably encompassed both these regions. It would be of interest to see whether the use of more restricted lesions might allow selective effects on *a* and *δ* to be achieved.

The STN is an important relay in the indirect striatofugal pathways, receiving input from various regions of the corpus striatum, including the AcbC, via the external pallidum; there are also reciprocal connections between the STN and parts of the cortex including the orbital prefrontal cortex (OPFC) [22]. It is therefore of interest to compare the effects of STN lesions seen here with previous studies of the effects of lesions of structures with connections to the STN. We recently examined the effects of lesions of the AcbC on the parameters of Eq. (1) [16]. The pattern of effect differed from the effects of STN lesions seen in the present experiment. Destruction of the AcbC increased the response time parameter *δ*, an effect also seen in the present experiment; however, unlike the present findings with STN lesions, AcbC lesions had no effect on the activation parameter *a* [16]*.* This suggests that, unlike the STN, the AcbC may not be involved in determining the instantaneous values of food reinforcers [16]. Lesions of the OPFC were found to reduce *a*, indicating a reduction of the incentive value of food reinforcers [29]. The opposite effects of STN and OPFC lesions on incentive value stand in contrast to the remarkable similarity of the effects of lesions of these two structures on some other behaviours, including facilitation of premature responding on the five-choice serial reaction time task and impairment of autoshaping [52]. It remains to be determined whether the effects of STN lesions seen in this experiment are attributable to disruption of functional connections between the STN and cortical and striatal regions. It will therefore be of interest, in future experiments, to examine the effect of disconnecting the STN from cortical and striatal regions on the parameters of Eq. (1).

In conclusion, this experiment employed a quantitative analysis of responding on progressive-ratio schedules, derived from Killeen’s [30] mathematical model of schedule-controlled behaviour, MPR. The present results support the notion that destruction of the STN enhances the incentive value of food rewards [11,48,49]. This in turn suggests that the STN may exert some limiting or ‘dampening’ influence over reinforcer value [48,49].

## Figures and Tables

**Table 1:** Estimated parameters of Eq. (1), goodness of fit (*r*^*2*^) and numbers of data points excluded from the function-fitting (mean ± SEM) of the STN-lesioned and sham-lesioned groups on three phases of the experiment (see text for explanation).

Parameter	Sham-lesioned group	STN-lesioned group
‘Specific activation’, *a* (s)*		
Phase 1 (one pellet)	149.8 ± 19.9	201.6 ± 37.6
Phase 2 (two pellets)	204.7 ± 25.0	308.4 ± 33.2
Phase 3 (one pellet)	133.0 ± 21.0	186.5 ± 29.7

‘Response time’, *δ* (s)#		
Phase 1 (one pellet)	1.09 ± 0.09	1.41 ± 0.12
Phase 2 (two pellets)	1.14 ± 0.09	1.51 ± 0.13
Phase 3 (one pellet)	0.99 ± 0.06	1.35 ± 0.12

‘Currency parameter’, *β*		
Phase 1 (one pellet)	0.40 ± 0.08	0.54 ± 0.10
Phase 2 (two pellets)	0.41 ± 0.10	0.38 ± 0.10
Phase 3 (one pellet)	0.59 ± 0.09	0.59 ± 0.11

Goodness of fit, *r*^2^		
Phase 1 (one pellet)	0.88 ± 0.01	0.83 ± 0.02
Phase 2 (two pellets)	0.72 ± 0.06	0.70 ± 0.05
Phase 3 (one pellet)	0.79 ± 0.03	0.75 ± 0.04

Number of data points excluded		
Phase 1 (one pellet)	0.4 ± 0.2	0.6 ± 0.2
Phase 2 (two pellets)	0.4 ± 0.2	0.1 ± 0.1
Phase 3 (one pellet)	0.3 ± 0.2	0.1 ± 0.1

* Significant effects of group and phase (*P* < 0.05), no significant interaction.

# Significant effect of group (*P* < 0.05), no significant of phase and no significant interaction.
